# Negative emotion evoked by viewing snakes has a motivating effect on cognitive processing in human children with or without intellectual disability

**DOI:** 10.1002/brb3.715

**Published:** 2017-05-02

**Authors:** Nobuo Masataka

**Affiliations:** ^1^ Primate Research InstituteKyoto UniversityInuyamaAichiJapan

**Keywords:** cognitive processing, down syndrome, emotion, intellectual disability, snake fear, survival

## Abstract

**Background:**

It is well known that prioritization of the processing of threatening stimuli generally induces deleterious effects on task performance. However, a study recently reported that emotion (possibly fear) evoked by viewing images of snakes exerts a facilitating effect upon making judgments of the images’ color in neurotypical adults and schoolchildren. Here, the author has attempted to confirm the relevance of this notion in children with and without intellectual disability.

**Methods:**

The author here compared the reaction time required to name the colors of snake and flower images between children with Down syndrome (DS) and mental age matched, typically‐developing (TD) children.

**Results:**

Snake images were responded to faster than flower images in both the groups, while the children with DS tended to respond more slowly overall.

**Conclusions:**

As in TD children, negative emotion can have a motivating effect on cognitive processing in children with DS. Some implications of the findings are pointed out with respect to the lower‐level task persistence as a characteristic motivational orientation in children with DS.

## 1 Introduction

It is well known that prioritization of the processing of threatening stimuli induces deleterious effects on the performance of tasks concerning the stimuli (Cohen, Dunbar, & McClelland, 1990; Ishai, Pessoa, Bikle, & Ungerleider, 2004; MacLeod, 2001; Öhman, Lundqvist, & Esteves, 2001). Asserting the opposite notion, Charles Darwin (1872) claimed that “a man or animal driven through terror of desperation, is endowed with wonderful strength” and argued that these responses are adaptive because men and animals possessing them are more adept at avoidance when exposed to evolutionarily dangerous stimuli. Recently, Darwin's argument was experimentally confirmed by a study (Shibasaki, Isomura, & Masataka, 2014) in which 108 adults and 25 children were required to name the color of images of snakes and flowers (Constantine, McNally, & Hornig, 2001). The reaction time (RT) to name the color of each stimulus was found to be shorter when snake images were presented as compared to when flower images were presented in both adults and children. Thus, the emotion (possibly fear) evoked by viewing an image of snakes as a biologically relevant threatening stimulus was likely to exert a facilitating rather than an interfering effect on making judgments of the snakes’ color. Here, the author reports that this effect was confirmed as well in a population of children with intellectual disability due to a known genetic etiology, Down syndrome (DS).

Intellectual disability is characterized by “impairment of skills manifested during the developmental period,” which includes “cognitive, language, motor and social abilities (World Health Organization, 1992).” While the mortality rate is higher in people with intellectual disability than in the general population (Glasson et al., 2002; Tyrer, Smith, McGrother, & Taub, 2007) research into factors associated with their survival is limited, especially from evolutionary and psychobiological perspectives. In order to address the question of such factors, the current study was undertaken. The results presented here indicate that an emotional‐cognitive association that appears likely to be crucial for the survival of humans (by enabling them to actively avoid evolutionarily relevant threatening stimuli) is preserved even in humans who are intellectually disabled.

DS is the most common genetic cause of intellectual disability. It occurs when an extra copy of chromosome 21 (or critical regions of chromosome 21) is present, producing a well‐recognized phenotype that includes characteristic facial and musculoskeletal features, increased risk for a number of health problems, and intellectual impairment (Baird & Sadvnik, 1987). The cognitive profile of individuals with DS is characterized by impaired executive functioning that includes inhibition‐related processes (Borella, Carretti, & Lanfranchi, 2012; Rowe, Lavender, & Turk, 2006).

The emotional profile of people with DS is also atypical. Generally, they are characterized as being cheerful and sociable (Hippolyte, Iglesias, & Barisnikov, 2009). Most studies of children with DS have shown impairments in recognition of negative emotional expressions, such as anger and fear (Kasari, Freeman, & Hughes, 2001). Adults with DS showed a positive bias when judging facial expressions and intensity of emotional expressions (Hippoyte, Barisnikov, & Van den Linden, 2008). Despite their ability to recognize positive and negative emotions, such as joy and sadness, they were likely to assess expressions as being more positive than they actually were. Overall, the reported studies seem to suggest that individuals with DS would be relatively unresponsive to danger signals. However, virtually all of the previous studies dealing with this issue have explored the social domain, using social signals such as facial expressions. In contrast, the author conducted this study using nonsocial and evolutionarily relevant danger signals.

### 1.1 The current study

If fear evoked by viewing snakes facilitates perception of the animals in individuals with DS, as it is known to do in neurotypical individuals (Shibasaki et al., 2014), then naming the color of snakes’ images should also be facilitated in individuals with DS as well. Such facilitation would not be observed if the unresponsiveness of individuals with DS to social danger signals extends to nonsocial danger signals, as represented by snakes. To address whether fear evoked by viewing snakes facilitates perception of the animals in individuals with DS, here the author compared the performance of the color‐naming of snake and flower images between youths with DS and typically‐developing (TD) children whose mental ages were matched to those of the youths with DS.

## 2 Methods

### 2.1 Participants

A group of 20 youths with DS (12 males and 8 females) aged 17–19 years (*M* = 18.40; range = 17.05–19.58) and 20 TD children (9 males and 11 females) aged 8 to 9 years (*M* = 8.83; range = 8.26–9.47) were tested in this study. All were Japanese. The TD children were individually matched to the DS participants, all of whom had a moderate intellectual disability, for gender and mental age. They were all right‐handed and naïve as to the purpose of this study and had normal or corrected‐to‐normal vision. They did not have any difficulty in color sensing.

All DS participants had a medical diagnosis of Trisomy 21. Significant sensory, psychiatric, or physical disabilities, as well as clinical symptoms of dementia, constituted exclusion criteria for participation.

The mental age of the TD children ranged from 7 to 10 years (*M* = 8.43; *SD* = 1.4). Full‐scale IQ scores ranged from 90 to 115 (*M* = 105.3; *SD* = 6.4). These children were recruited via the board of education in a small city in Japan. All of them attended normal classes corresponding to their chronological age level. Teachers of the classes were asked to choose these children from the average level of their classes so that inclusion of advanced or delayed children relative to their ages could be avoided.

### 2.2 Procedure

As the stimuli presented in the experiment, 20 photographs of snakes and 20 photographs of flowers were prepared. Each of the 40 photographs appeared three times – once in red, once in green, and once in blue – for a total of 120 experimental trials. To create color‐filtered images, we first removed all color content from the photographs, generating black and white images. Subsequent color balance manipulation along three different dimensions transformed the shadows, midtones, and highlights of each grayscale image. Each stimulus was presented against a black background.

A 22‐inch monitor connected to a personal computer was placed on a table. An adapted single‐trial version of the pictorial emotional Stroop task (Constantine et al., 2001; Shibasaki et al., 2014; 9 was used in the present experiment. Participants were told by an experimenter, who had not been notified about the purpose of this study, that they would see a series of color‐filtered images, and they were instructed to indicate the color of each image as quickly as possible via a key‐press, while ignoring the content of each image. During the experiment, they were required to put the index, the middle and the third finger of the right hand upon three different keys on an external numeric keypad, and were instructed to press the index finger to indicate that the picture was red, to press the middle finger to indicate that the picture was green, and to press the third finger to indicate that the picture was blue. The relationship between keypad position and color was counterbalanced across the participants. More detailed information about the protocol has been provided elsewhere (Shibasaki et al., 2014).

## 3 Results

**Figure 1:**
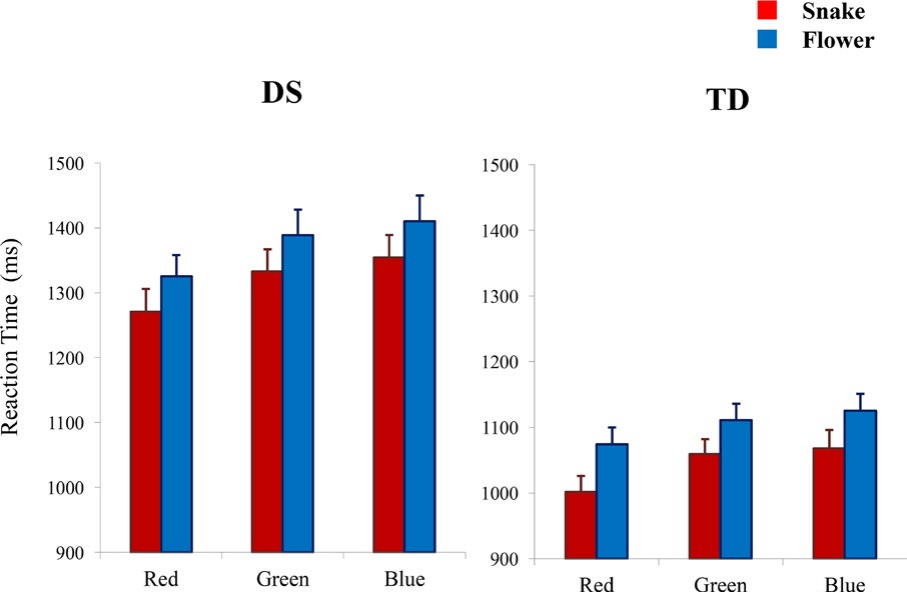
Reaction times of the participants in the Stroop task. Left: youths with DS, right: TD children

The overall results of the experiment are summarized in Figure 1. When the collected data were analyzed using a 2 (participant group, PARTICIPANT) × 2 (snake/flower, IMAGE) × 3 (red/green/blue, COLOR) ANOVA, all of the three main effects were statistically significant (*F*_1,35_ = 44.50, *p* = .0000,$\eta_{p}^{2}$ = 0.528 for PARTICIPANT, *F*_1,35_ = 34.92, *p* = .0000, $\eta_{p}^{2}$ = 0.482 for IMAGE, *F*_2,70_ = 14.62, *p* = .0000, $\eta_{p}^{2}$ = 0.295 for COLOR). The interaction between PARTICIPANT and IMAGE was not significant (*F*_1,35_ = 0.44, *p* = .36, $\eta_{p}^{2}$ = 0.023). There was no significant interaction between PARTICIPANT and COLOR (*F*_2,70_ = 0.95, *p* = .34, $\eta_{p}^{2}$ = 0.026). The interaction between IMAGE and COLOR was not significant (*F*_2,70_ = 0.47, *p* = .63, $\eta_{p}^{2}$ = 0.013). The interaction among PARTICIPANT, IMAGE and COLOR was also not significant (*F*_2,70_ = 0.60, *p* = .55, $\eta_{p}^{2}$ = 0.017).

## 4 Discussion

For the youths with DS, the present results showed overall slower RTs than those of the TD children across all of the materials. This finding is consistent with previous findings of the basic slower processing speed of individuals with DS in comparison with control groups (Chapman & Herketh, 2000; Rowe et al., 2006; Silverman, 2007). This probably more or less reflects some limitations of underlying cognitive mechanisms (probably limitations of inhibitory processing) of the youths with DS, and appears to be closely related to the higher rate of mortality in individuals of this population (Baird & Sadvnik, 1987; Tyrer et al., 2007) under natural circumstances.

In both the youths with DS and the TD children, however, RTs to name the color of red images were shorter when compared with those for naming the color of green or blue images. These findings about the relationship between RTs and the color of stimuli were predictable because accumulating evidence has indicated that a stimulus presented in red draw attention more readily than the same stimulus presented in other colors, such as green or blue (Shibasaki et al., 2014).

More important, both the youths with DS and the TD children were faster in processing snake images than flower images, indicating the fact that an equivalent motivating effect evoked by viewing snakes on their cognitive processing was confirmed in the youths with intellectual disability due to Down syndrome. Given their slower processing speed, the youths with DS would be predicted to be less adept at avoiding snakes than TD children. Nevertheless, the degree to which the processing of snake images was facilitated per se was not different according to whether the participants had or did not have DS. Thus, snake images as evolutionarily relevant threatening stimuli should have evoked negative emotion in individuals with DS as well, which, in turn, should have enhanced processing of the stimuli in them as in TD individuals. The emotion‐cognition association underlying such enhancement appears not to be impaired in individuals with DS, probably because this association is a fundamental mechanism which would enable them to survive when exposed to various dangers under natural circumstances.

Recent research indicates that a similar motivating effect of negative emotion can also be demonstrated in the social domain, that is, with regard to threatening and fearful facial expressions (Phelps, Ling, & Carrasco, 2006), but individuals with DS have been reported to be insensitive to such variations of facial expressions (Hippoyte et al., 2008; Kasari et al., 2001). This insensitivity suggests the possibility that a strong motivational effect is not exerted in them when interacting with someone who is displaying such expressions. This may be related to the personality‐motivational style characteristic of individuals with DS, namely, being cheerful and sociable emotionally, with less persistence and more distractibility than usual with respect to task performance (Pitcairn & Wishart, 1994; Viachou & Farrell, 2000).

In all, this study demonstrates that the threat superiority in the nonsocial domain persisted in youths with DS despite their intellectual disability, though the study alone does not tell whether similar pattern of threat superiority can be obtained in other conditions with intellectual disability such as severe Kanner autism. Another finding obtained from the results of the study is that youths with DS responded to snake and flower images drastically more slowly than matched‐controls, though whether this effect is related to the intellectual disability per se, or to some other symptoms of DS is still unknown. One way to examine that is to see if the group difference persists after controlling for the difference of IQ. Apparently, these are issues to be investigated in the near future.

## Conflict of Interest

None declared.
